# Effect of photobiomodulation on trapezius myofascial pain: a randomized, sham-controlled, double-blind clinical trial

**DOI:** 10.1007/s10103-026-04964-8

**Published:** 2026-07-27

**Authors:** Rayanne Kethleen do Nascimento Silva, Andreza Maria Macau Farias Rocha, Valéria Mayaly Alves de Oliveira, Juberlânia do Nascimento Matias dos Santos, José Diego Sales do Nascimento, Palloma Rodrigues de Andrade

**Affiliations:** 1 Postgraduate Program in Physical Therapy, Federal University of Paraíba, João Pessoa, Brazil; 2 Department of Physical Therapy, Federal University of Paraíba, João Pessoa, Brazil

**Keywords:** Chronic pain, Trigger points, Low-level laser therapy

## Abstract

To investigate the effect of 16 J of 808 nm low-level laser therapy (LLLT) on pain in individuals with trapezius trigger points (TPs). Randomized, double-blind, controlled trial conducted with 52 adults (≥ 18 years) with chronic trapezius pain and TPs. Participants were assigned to the LLLT group (808 nm, 16 J per TP) or the sham group (same protocol without emission). Both received eight sessions over four weeks. Outcomes included pain perception, number of TPs, cervical function, skin temperature, global perceived effect, and adverse events. Randomization was concealed from evaluators and participants. Repeated measures ANOVA (SPSS 21) was used for analysis. No significant differences were found between groups for pain [F(2,100) = 1.21; p = 0.30, η2 = 0.024], TPs [F(1.09,54.27) = 0.35; p = 0.57, η2 = 0.007], cervical skin temperature [F(1.79,89.65) = 0.79; p = 0.45, η2 = 0.042], trapezius temperature [F(2,100) = 1.09; p = 0.34, η2 = 0.021], or global perceived effect [F(1.44) = 1.91; p = 0.17, η2 = 0.029]. Cervical function improved significantly in the LLLT group [F(1.36,67.73) = 3.75; p = 0.04, η2 = 0.070]. Adverse effects were mild and infrequent, mainly warmth. Both groups reported clinically relevant pain reduction. LLLT at 16 J per TP with 808 nm was not superior to placebo for pain, TP count, or skin temperature. However, it improved cervical function, suggesting a functional benefit. Registration: july 18, 2022 on the Brazilian Clinical Trials Registry (REBEC – RBR-10m474jv).

## Introduction

Myofascial pain syndrome (MPS) is a musculoskeletal condition that can cause intense, deep pain in muscles and fascia [1], resulting from inflammation or alterations in the relationship between muscles and their surrounding structures. It is described as a specific type of persistent pain syndrome that may be accompanied by significant emotional dysfunctions or abnormalities and recurs in the muscles, fascia, or other soft tissues [2]. This condition is highly prevalent across all ages, ethnicities, and cultures [3]. More than 55% of neck pain cases may be associated with this syndrome [4], and it represents a leading cause of work incapacity among middle-aged individuals [2].

The main characteristic of MPS is the presence of trigger points (TPs), which are hyperirritable spots located within a palpable taut band of skeletal muscle fibers that produce both local and referred pain [1–3]. TPs are classified as active or latent. Active TPs are spontaneously painful, whereas latent TPs are only painful upon palpation, making their identification more challenging in clinical practice [5–6]. The diagnostic criteria for TPs typically include a minimum of two out of three signs: taut band, hypersensitive spot, and referred pain [6].

MPS in the neck region is associated with a high frequency of TPs in the trapezius muscle [8]. TPs are concentrated in well-defined areas, approximately 10 mm from the innervation zone in the middle third of the trapezius muscle [9], and their detection is relatively easy for experienced physiotherapists [10]. The presence of TPs in the trapezius muscle can compromise the functional capacity of individuals with neck pain [5, 7]. Additionally, TPs may affect the skin temperature (Tsk) of the region due to inflammatory processes or local microcirculatory compression. Therefore, infrared thermography appears to provide an objective measure for monitoring treatment effectiveness [7].

Photobiomodulation stands out as an intervention capable of modulating the inflammatory process and enhancing local microcirculation, thereby contributing to the management of TPs [11]. Low-level laser therapy (LLLT) using near-infrared wavelengths (approximately 800–830 nm) has been widely applied as a photobiomodulation approach for musculoskeletal pain relief [12]. However, although physiological mechanisms support its use, the literature still presents heterogeneous and inconclusive evidence regarding the most effective dosage for treating MPS, particularly in regions such as the cervical area and upper trapezius [13].

For example, while some studies recommend doses ranging from 0.24 J to 18 J [14–16], the World Association for Photobiomodulation Therapy-WALT [17] recommends dosimetries of up to 16 J for nonspecific musculoskeletal pain in the spine. Therefore, as systematic reviews have not established optimal dosimetry parameters [12, 16], there is no consensus regarding the ideal dose of LLLT for this condition. Particularly with respect to higher energy levels, such as the 16 J recommended by WALT for the treatment of TPs in the trapezius region, clinical trials remain scarce.

In this context, the aim of this study was to investigate the effectiveness of LLLT at 808 nm and 16 J per trigger point on pain intensity, neck disability, skin temperature, and global perceived effect in individuals with myofascial trigger points in the upper trapezius muscle.

## Methods

This study was a randomized, sham-controlled, double-blind, superiority clinical trial conducted at the Laboratory of Studies in Dermatofunctional Physical Therapy, Federal University of Paraíba (UFPB), after approval by the Ethics Committee of the Health Sciences Center (53667221.8.0000.5188) and prospective (july 18, 2022) registration on the Brazilian Clinical Trials Registry (REBEC– RBR-10m474jv- Effect of photobiomodulation application on neck muscle pain**).**

The study was conducted in accordance with the ethical principles established in the Declaration of Helsinki, and all participants voluntarily signed an Informed Consent Form (ICF). The trial report followed the recommendations outlined in the Consolidated Standards of Reporting Trials [18]. There was no public involvement in the research.

### Sample, randomization, allocation, and blinding

The sample included male and female participants aged 18 years or older who presented spontaneous pain (active TP) in the trapezius region lasting more than 3 months, with a confirmed diagnosis of myofascial trigger points in this area according to Delphi-based criteria of Fernandes-De-Las-Peñas and Dommerholt [6]. Participants could not be undergoing any other physical therapy treatment or using psychotropic or anti-inflammatory medications during the study period. Recruitment occurred through social media advertisements and referrals from the Physical Therapy School Clinic at UFPB.

Sample size was calculated a priori using G*Power 3.1 software, based on a 10-point pain intensity scale and an effect size of 0.21 derived from a previous study [19]. The calculation considered a statistical power of 90%, α ≤ 0.05, a correlation coefficient of 0.5, two groups, three measurements, and a potential 20% dropout rate, resulting in a required sample size of 52 participants, equally allocated between the two groups (*n* = 26 per group).

After screening and assessment, participants were randomly allocated (1:1 ratio) via www.randomizer.org into two parallel groups: the Low-Level Laser Therapy group (LLLTG) and the sham group (SHAMG). Randomization was performed and kept confidential by an external researcher to ensure both the evaluator and participants were blinded to group allocation.

### Outcome measures

All outcomes were assessed by a single blinded evaluator who was properly trained and had at least three years of experience in the use of the instruments and in the assessment procedures. Pain perception, TPs number, neck disability, and skin temperature (Tsk) in neck and trapezius regions were assessed at three time points: before the intervention (T0), immediately after the last treatment session (T1), and 48 h after the final session (T2). Additionally, at T1 and T2, the perceived overall effect and potential adverse effects were evaluated.

Pain intensity was measured using the Numeric Pain Rating Scale (NPRS), where 0 represents “no pain” and 10 “the worst pain imaginable” [20]. The Minimal Clinically Important Difference (MCID) for patients with cervical pain without radicular symptoms is 1.5 points [21]. Cervical functionality was evaluated using the Neck Disability Index (NDI), which assesses the impact of cervical pain on activities of daily living through 10 questions related to general activity and pain [22]. The MCID for nonspecific neck pain is 5.5 points [21].

Tsk was measured using infrared thermography. Thermograms were captured in a climate-controlled environment (22.94 ± 1.34 °C; 42.18 ± 7.51% relative humidity), monitored by a digital meteorological station (WMR86, Oregon Scientific, China). Participants were instructed to fast for two hours before image capture, avoid stimulants, hot showers, cosmetics, medications, physical activity, or friction on the neck and trapezius region for 48 h prior to assessment [23].

**Fig. 1:**
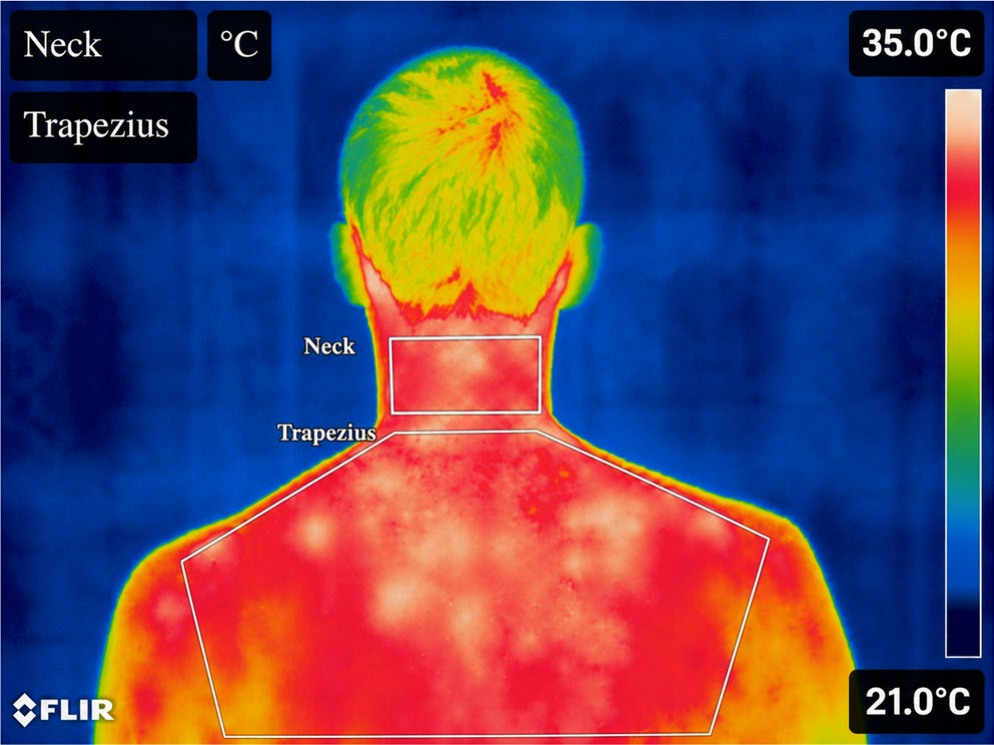
Regions of interest in infrared thermography

A FLIR E54 camera (FLIR Systems, USA) with IR resolution of 320 × 240 pixels, temperature range of − 20 °C to 120 °C, thermal sensitivity < 40 mK @ 30 °C, and accuracy ± 2% was used to capture cervical and trapezius thermal profiles. The camera was positioned 90° to the region of interest (ROI) on a tripod 1.5 m away, with emissivity set at 0.98 and reflected temperature at 20 °C. After a 15-minute acclimatization period, images were captured and processed using FLIR Ignite (Teledyne FLIR). Two ROIs were defined for temperature analysis: one for the cervical region and another over the trapezius (Fig. 1).

Trigger points were diagnosed according to Delphi-based consensus criteria [6], whereby the presence of all essential (core) diagnostic features was required. Specifically, a diagnosis was established only when two manual findings (a palpable taut band and a hypersensitive spot) and one clinical symptom (deep, referred pain) were simultaneously identified. No additional complementary diagnostic features were considered mandatory for inclusion. Palpation of the trapezius muscle was performed by an experienced physiotherapist, with participants positioned in the prone position, the cervical spine in a neutral position, shoulders relaxed, and arms extended alongside the body. All TPs in the trapezius muscle were identified and quantified (both active and latent).

**Fig. 2:**
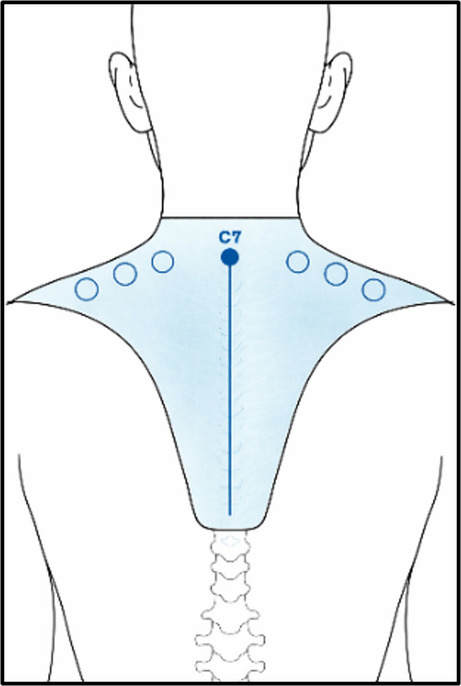
Delimitation of the trapezius treatment area

TPs locations were marked on an acetate sheet template (Fig. 2) to ensure that the same points were treated throughout the protocol. The template consisted of an acetate material positioned over the trapezius area. The spinous process of the seventh cervical vertebra (C7) was used as the central anatomical reference point. From this reference, manual palpation was performed to identify and mark myofascial trigger points. Each participant had a customized template due to individual variability in TPs distribution. This procedure was carried out to ensure accurate identification and treatment of all TPs. Apley’s compression and distraction tests were used to rule out radicular pain due to nerve injury.

The Global Perceived Effect (GPE) was assessed using a numerical scale from − 5 to + 5, where − 5 indicated extreme worsening and + 5 complete recovery [24]. Adverse effects were recorded at the end of the last treatment session, asking participants if they experienced any sensations related to the LLLT protocol; if so, they were asked to describe and rate the intensity (mild, moderate, or severe).

### Intervention procedures

After the initial assessment (T0), performed by a researcher blinded to participant allocation, all participants underwent the intervention procedures administered by a physical therapist. Each participant received the intervention protocol corresponding to their allocation group over a 4-week period, with 2 sessions per week on nonconsecutive days, totaling 8 sessions. Each TPs in the treated region received 160 s of irradiation or its simulation. During these procedures, participants were positioned prone, with relaxed shoulders and the cervical spine in a neutral position. Participants were reassessed immediately after completion of the intervention protocol and 48 h later (T1 and T2, respectively).

The LLLTG received point-by-point application of an 808 nm laser using the Therapy XT device (DMC, São Carlos, São Paulo, Brazil), equipped with a round-tipped probe (3.54 mm in diameter, corresponding to a spot area of 0.09842 cm²) and an effective output power of 100 mW. The probe was positioned perpendicularly to the treatment area, delivering a total energy of 16 J with an energy density of 162.6 J/cm² (Table 1). The total irradiation time required to deliver 16 J at each treated trigger point was 160 s.

**Table 1:** Irradiation parameters used in the photobiomodulation protocol

PARAMETER	SPECIFICATION
Source type	Low-level laser therapy (LLLT)
Equipment	Therapy XT (DMC Equipamentos)
Wavelength	808 nm ± 10 nm (infrared)
Output power	100 mW (0.1 W)
Spot área	0.09842 cm²
Irradiance	1.02 W/cm²
Energy per point	16 J
Time per point	160 s
Energy density	162.6 J/cm²
Emission mode	Continuous
Number of points	According to each participant’s assessment
Application site	Trapezius muscle
Application technique	Point application, in contact with the skin
Applied pressure	Light pressure
Number of sessions	8 sessions
Treatment interval	Twice a week on non-consecutive days

*nm *Nanometers, *mW *Milliwatts, *W *Watts, *cm*² Square centimeters, *W*/*cm*² Watts per square centimeter, *J *Joules *J*/*cm*² Joules per square centimeter, *s *Seconds

The SHAMG underwent the same procedure with the laser device turned on to emit sound cues, but without light emission, that is, without pressing the start button for emission. Participants wore protective goggles that completely blocked their vision, ensuring that they could only hear the sounds without seeing the procedure. The therapist simulated the laser application, spending the same duration of 160 s per treated TP. This procedure was performed to ensure that both participants and evaluators were blinded to allocation.

### Statistical analysis

All data were analyzed according to the intention-to-treat principle, with missing data imputed using baseline values, representing a conservative approach for handling loss to follow-up [25]. Statistical analyses were performed using SPSS v21. The primary outcome was pain intensity assessed using the NPRS. Secondary outcomes included neck disability (NDI), skin temperature assessed by infrared thermography, and GPE.

Categorical variables were expressed as absolute (N) and relative (%) frequencies, and numerical variables as mean ± standard deviation (SD). Normality and homogeneity were verified using Kolmogorov-Smirnov and Levene tests. Depending on Mauchly’s test of sphericity or Greenhouse-Geisser correction, between-group differences and 95% confidence intervals (95% CI) were calculated using two-way repeated measures ANOVA with Bonferroni post hoc tests for pain intensity, skin temperature, and cervical functionality. A two-way ANOVA was also applied to assess inferences related to the global perceived effect.

## Results

**Fig. 3:**
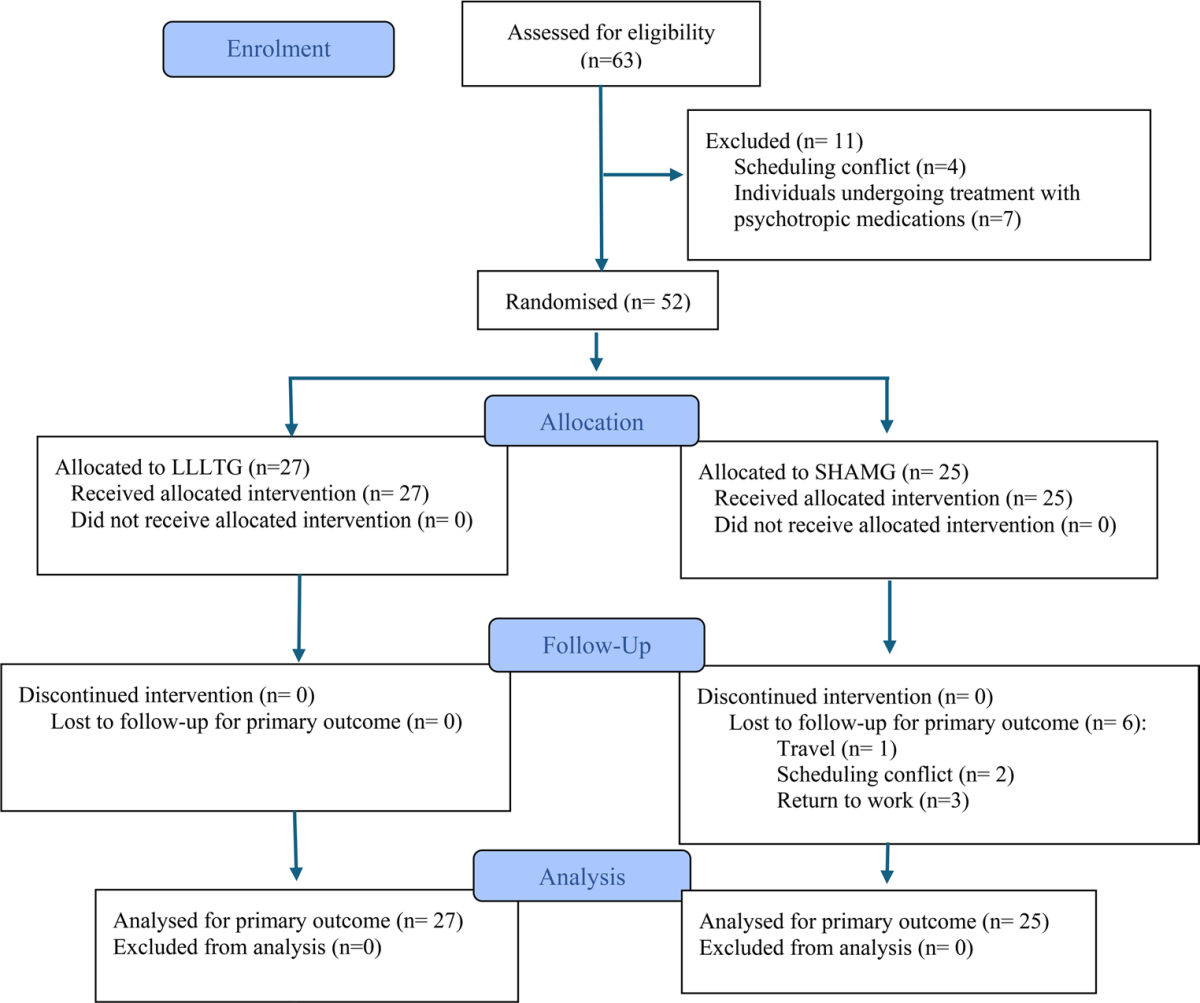
Sample flowchart

Initially, 63 volunteers were recruited by convenience sampling for eligibility screening; however, 11 were excluded for not meeting the inclusion criteria, such as the use of sedative or anxiolytic medications (*n* = 7) and scheduling conflicts (*n* = 4). The final sample consisted of 52 volunteers randomly allocated into two groups (LLLTG = 27 and SHAMG = 25). All participants agreed to take part in the study; however, in the SHAMG, six participants were lost to follow-up due to travel (*n* = 1), returning to work activities (*n* = 3), or incompatible schedules (*n* = 2). Nevertheless, their data were included in the analyses following the intention-to-treat principle (Fig. 3). The participants presented similar baseline characteristics, as shown in Table 2.

**Table 2:** Baseline characteristics of the volunteers

VARIABLES	mean ± SD			
	LLLTG	SHAMG		t (CI)
Age (years)	32.04 ± 15.12	30.16 ± 9.55	0.53 (3.54 to -5.23)	
Body mass (kg)	68.07 ± 15.24	70.60 ± 17.10	-0.56 (-11.54 to 6.48)	
Height (m)	1.66 ± 0.09	1.66 ± 0.09	-0.14 (-0.05 a 0.05)	
Pain intensity (0–10 points)	5.11 ± 1.45	5.48 ± 1.56	-0.88 (-1.21 to 0.47)	
Pain onset (years)	4.32 ± 4.24	3.51 ± 4.24	0.69 (-1.55 to 3.17)	
Cervical Tsk (°C)	32.61 ± 0.75	32.38 ± 0.74	1.08 (-0.19 to 0.64)	
Trapezius Tsk (°C)	32.02 ± 0.69	31.93 ± 0.93	0.37 (-0.37 to 0.54)	

*LLLTG *Low Level Laser Therapy Group, *SHAMG *Sham Group, *t *t-test value, *CI *Confidence interval, *SD *Standard deviation, *kg *Kilograms, *m *Meters, *°C* Degrees Celsius

The means and standard deviations of pain intensity (NPRS), number of TPs, neck disability (NDI), skin temperature (Tsk) in the neck and trapezius regions, and global perceived effect (GPE) are presented in Table 3.

**Table 3:** Mean and standard deviation of the variables by group and assessment time

VARIABLES	LLLTG			SHAMG		
	T0	T1	T2	T0	T1	T2
NPRS	5.11 ± 1.45	3.22 ± 2.42	2.33 ± 2.34	5.48 ± 1.56	3.00 ± 2.38	3.08 ± 2.93
Number of TPS	7.07 ± 1.88	4.04 ± 1.63	3.78 ± 1.60	6.68 ± 1.60	3.88 ± 2.37	3.76 ± 2.40
NDI	35.91 ± 17.54	20.64 ± 16.61	18.16 ± 14.64	36.76 ± 17.01	26.66 ± 18.94	28.76 ± 20.69
Cervical Tsk						
Maximum	33.51 ± 0.64	33.56 ± 0.57	33.59 ± 0.85	33.19 ± 0.66	33.47 ± 1.02	33.50 ± 0.90
Minimum	30.64 ± 1.75	30.74 ± 1.67	30.57 ± 1.82	30.92 ± 1.38	31.32 ± 1.47	31.38 ± 1.46
Mean	32.61 ± 0.75	32.62 ± 0.77	32.64 ± 0.98	32.38 ± 0.74	32.68 ± 1.25	32.71 ± 0.98
Trapezius Tsk						
Maximum	33.34 ± 0.63	33.20 ± 0.71	33.17 ± 0.79	33.04 ± 0.78	33.12 ± 1.11	33.38 ± 0.93
Minimum	30.58 ± 0.89	30.56 ± 1.04	30.54 ± 1.34	30.46 ± 1.09	30.58 ± 1.44	30.96 ± 1.31
Mean	32.02 ± 0.69	31.93 ± 0.91	31.99 ± 1.02	31.93 ± 0.93	31.96 ± 1.20	32.27 ± 1.07
GPE	---	2.70 ± 1.30	3.14 ± 1.39	---	2.95 ± 1.58	2.63 ± 1.46

T0- Baseline; T1- immediately after the therapeutic application; T2- 48 h after the last session

*NPRS *Numeric Pain Rating Scale, *TPs *Trigger Points, *NDI *Neck Disability Index, *GPE* Global Perceived Effect, *LLLTG *Low Level Laser Therapy Group, *SHAMS *Sham Group

No statistically significant differences were observed between groups for pain intensity (NPRS) [F(2,100) = 1.21; *p* = 0.30, η^2^ = 0.024] or the number of trigger points [F(1.09, 54.27) = 0.35; *p* = 0.57, η^2^ = 0.007]. On the other hand, a significant difference was found in the Neck Disability Index (NDI) [F(1.36, 67.73) = 3.75; *p* = 0.04, η^2^ = 0.070], indicating that the group treated with LLLT showed greater functional improvement compared with the SHAMG (Table 4).

**Table 4:** Comparison of mean pain, number of trigger points, cervical region functionality, and skin temperature (mean difference and 95% CI)

VARIABLES	LLLTG			SHAMG			LLLTG vs. SHAMG		
	INTRAGROUP			INTRAGROUP			T0	T1	T2
T0 vs. T1	T0 vs. T2	T1 vs. T2	T0 vs. T1	T0 vs. T2	T1 vs. T2				
NPRS	1.89* (0.78 to 2.99)	2.78* (1.70 to 3.86)	0.89 (-0.16 to 1.93)	2.48* (1.33 to 3.63)	2.40* (1.28 to 3.52)	-0.08 (-1.17 to 1.01)	-0.37 (-1.21 to 0.47)	0.22 (-1.12 to 1.56)	-0.75 (-2.22 to 0.72)
Number of TPs	3.04* (2.13 to 3.95)	3.30* (2.33 to 4.27)	0.26* (0.03 to 0.49)	2.80* (1.85 to 3.75)	2.92* (1.91 to 3.93)	0.12 (-0.12 to 0.36)	0.39 (-0.58 to 1.37)	0.16 (-0.97 to 1.28)	0.02 (-1.11 to 1.15)
NDI	15.27* (8.20 to 22.34)	17.75* (10.92 to 24.88)	2.48 (-0.93 to 5.89)	10.09* (2.74 to 17.44)	7.99* (0.58 to 15.41)	-2.10 (-5.64 to 1.45)	-0.85 (-10.48 to 8.79)	-6.03 (-15.93 to 3.88)	-10.60* (-20.53 to -0.68)
Cervical Tsk									
Maximum	-0.05 (-0.44 to 0.34)	-0.07 (-0.43 to 0.29)	-0.03 (-0.46 to 0.41)	-0.28 (-0.68 to 0.13)	-0.31 (-0.69 to 0.06)	-0.04 (-0.49 to 0.42)	0.32 (-0.04 to 0.68)	0.91 (-0.36 to 0.55)	0.81 (-0.41 to 0.57)
Minimum	-0.09 (-0.98 to 0.80)	0.07 (-0.57 to 0.72)	0.17 (-0.78 to 1.11)	-0.40 (-1.32 to 0.53)	-0.46 (-1.14 to 0.21)	-0.07 (-1.05 to 0.91)	-0.28 (-1.16 to 0.61)	-0.58 (-1.46 to 0.30)	-0.81 (-1.74 to 0.11)
Mean	-0.02 (-0.49 to 0.46)	-0.03 (-0.40 to 0.33)	-0.02 (-0.51 to 0.48)	-0.29 (-0.79 to 0.21)	-0.32 (-0.70 to 0.06)	-0.03 (-0.55 to 0.48)	0.22 (-0.19 to 0.64)	-0.05 (-0.63 to 0.52)	-0.07 (-0.61 to 0.48)
Trapezius Tsk									
Maximum	0.14 (-0.31 to 0.60)	-0.17 (-0.18 to 0.52)	0.03 (-0.39 to 0.45)	-0.08 (-0.55 to 0.40)	-0.34 (-0.70 to 0.03)	-0.26 (-0.70 to 0.18)	0.30 (-0.09 to 0.69)	0.08 (-0.43 to 0.60)	-0.21 (-0.69 to 0.27)
Minimum	0.02 (-0.49 to 0.54)	0.05 (-0.47 to 0.57)	0.02 (-0.55 to 0.60)	-0.12 (-0.66 to 0.42)	-0.50 (-1.04 to 0.04)	-0.38 (-0.97 to 0.22)	0.12 (-0.43 to 0.67)	-0.03 (-0.72 to 0.67)	-0.43 (-1.17 to 0.32)
Meam	0.09 (-0.35 to 0.53)	0.02 (-0.36 to 0.41)	-0.07 (-0.52 to 0.39)	-0.02 (-0.48 to 0.43)	-0.34 (-0.73 to 0.06)	-0.31 (-0.79 to 0.16)	0.08 (-0.37 to 0.54)	-0.03 (-0.62 to 0.56)	-0.28 (-0.86 to 0.31)
GPE	---	---	-0.37 (-0.82 to 0.08)	---	---	0.11 (-0.43 to 0.64)	---	-0.24 (-1.10 to 0.61)	0.23 (-0.68 to 1.14)

T0- Baseline; T1- immediately after the therapeutic application; T2- 48 h after the last session

*NPRS* Numeric Pain Rating Scale, *TPs *Trigger Points, *NDI *Neck Disability Index, *GPE *Global Perceived Effect, *LLLTG *Low Level Laser Therapy Group, *SHAMS *Sham Group

*-*p* < 0.05

No statistically significant differences were observed in maximum, mean, or minimum skin temperature in either the cervical or trapezius regions over time. In the cervical region, the results were: maximum temperature [F(2,100) = 0.68; *p* = 0.51, η^2^ = 0.013], mean temperature [F(1.79, 89.65) = 0.79; *p* = 0.45, η^2^ = 0.042], and minimum temperature [F(1.71, 85.69) = 0.61; *p* = 0.52, η^2^ = 0.012]. In the trapezius region, the values were: maximum temperature [F(2,100) = 2.28; *p* = 0.11, η^2^ = 0.044], mean temperature [F(2,100) = 1.09; *p* = 0.34, η^2^ = 0.021], and minimum temperature [F(2,100) = 1.61; *p* = 0.20, η^2^ = 0.031] (Table 4).

The comparison of the Global Perceived Effect (GPE) between the LLLTG and SHAMG groups at T1 and T2 showed no statistically significant difference over the follow-up period [F(1,44) = 1.91; *p* = 0.17, η^2^ = 0.029] (Table 4), indicating a similar perception of clinical improvement among participants in both groups.

Only 18.5% of participants in the LLLTG and 8% in the SHAMG reported any adverse effects. Among the reported effects, a warming sensation was the most frequent in the LLLTG (14.8%), while in the SHAMG only 4% of participants reported this sensation. A tingling sensation was mentioned by 3.7% of participants in the LLLTG but was not reported in the SHAMG. Conversely, 4% of participants in the SHAMG reported headaches, an effect not observed in the LLLTG. Moreover, all participants who reported any adverse effects described the intensity as mild.

## Discussion

Based on the data analysis, it was observed that: (1) there were no statistically significant differences in pain intensity, number of TPs, or skin temperature (Tsk) in the trapezius region, nor in global perceived effect, between the intervention groups; (2) a significant improvement in cervical functionality was found in the group treated with LLLT compared with the SHAMG, suggesting a possible functional benefit of the intervention; and (3) a low frequency and intensity of reported adverse effects, reinforcing the safety profile of LLLT when applied with the parameters used in this study.

These findings may be explained by factors such as the natural history of the condition, the therapeutic window adopted in this study, and the characteristics of pain induced by TPs. Regarding the natural history of chronic neck pain, it is known to fluctuate over time, with spontaneous improvement or worsening without a linear progression, regardless of the intervention applied [26].

The efficacy of LLLT appears to depend directly on variables such as dosage, frequency, and number of sessions [27]. Applications using wavelengths between 820 and 830 nm and doses between 0.8 and 9 J per point seem to produce better effects on neck pain [16]. However, as these parameters are not yet fully established and WALT does not define them precisely in its guidelines, dosimetry for TP-related pain remains highly variable.

LLLT has demonstrated evidence of analgesic effects through different pain modulation mechanisms, although these effects may be less evident in chronic conditions. This may occur because MPS can present nociceptive and nociplastic characteristics depending on individual variability [28], making its treatment a challenge in clinical practice. In a meta-analysis including 13 studies with 556 patients, it was demonstrated by Tehrani et al. [29] that the efficacy of LLLT in myofascial pain syndrome is considerably lower than in other musculoskeletal disorders.

No significant reduction in the number of TPs was observed between groups, similar to the findings for pain intensity. It was expected, however, that individuals treated with LLLT would show a significantly lower number of TPs at the end of the protocol. This expectation was based on studies suggesting that LLLT promotes cellular and tissue changes by regulating cellular metabolism, improving blood circulation, and consequently minimizing local hypoxia [27], factors considered relevant in the pathophysiology of TP formation. Nonetheless, the findings of this study do not support this hypothesis. Previous systematic reviews have demonstrated that, although statistically significant, the reduction in neck pain in patients with myofascial dysfunction is not clinically meaningful [29, 30].

A possible explanation for this result may lie in the energy dosage used (16 J per point) applied to the TPs in this study. Considering the irradiation time and application area, it is possible that the energy level exceeded the optimal range for photobiomodulation effects on TPs. According to the Arndt–Schulz law, excessively low doses tend to be ineffective, whereas overly high stimuli may trigger inhibitory physiological responses [31], which could have influenced the lack of therapeutic responses for both pain and TP reduction. In this context, a high total energy dose combined with parameters such as wavelength and output power may lead to undesirable effects, such as impairing the healing process [32]. Although the present study followed WALT criteria [31], in which higher-energy doses and longer application times are suggested for lasers with higher output powers, the selected energy did not reduce pain or the number of TPs. Thus, although this intensity proved safe due to the low frequency and mild nature of adverse effects, it was not effective for pain reduction or TP modulation.

Skin temperature (Tsk) also did not change throughout the treatment, consistent with the findings of Ferreira et al. [33], who did not identify changes in Tsk 48 h after a single laser session, although the region of interest (ROI) of the trapezius was evaluated after only one session. In contrast, the findings of Hakgüder et al. [7] demonstrated that LLLT application in patients with MPS reduced thermal asymmetry and temperature gradients between affected and unaffected sides, interpreted as a stabilizing effect on local inflammatory activity. In the present study, the TP area was not assessed in isolation to allow comparison with unaffected areas; instead, the entire region of interest (cervical and trapezius) was evaluated. This methodological difference may explain the discrepancy in findings. Furthermore, in that study, LLLT was combined with a stretching program, and isolated LLLT stimulation at the TP site may not have altered the mean Tsk in these regions. A more detailed analysis of the area surrounding the TP might clarify whether microcirculatory changes occurred, although this was not feasible with the imaging acquisition and analysis methods used in this study.

Regarding cervical functionality, the results corroborate a recent meta-analysis by Liu et al. [34], which different noninvasive therapies for MPS were compared, demonstrating that LLLT was more effective than control interventions in reducing pain-related functional disability (MD: −4.58; 95% CI: −7.80 to − 1.36). LLLT was compared with different power settings and parameters, and the quality of evidence ranged from moderate to very low, suggesting cautious interpretation. In this context, it is important to highlight that the present study was conducted with methodological rigor, including a randomized, placebo-controlled design with double blinding of both evaluators and participants. These methodological precautions strengthened the internal validity of the findings and minimized potential biases that could compromise study quality. Furthermore, the mean difference between LLLTG and SHAMG at T2 was − 10.6 points in NDI, which exceeds the Minimal Clinically Important Difference (MCID) of 5.5 for this instrument when applied to patients with nonspecific neck pain [21].

While the LLLTG was not superior to the SHAMG for pain outcomes after completion of the treatment protocol, the cumulative effects of LLLT appear to improve neck disability, demonstrating that pain and functional outcomes do not always respond in the same way [35]. Chronic cervical myofascial pain is multifactorial [36]. Several factors may have influenced these results, such as the non-treatment of latent TPs, as well as the lack of control of other factors, including forward head posture and shoulder protraction, and even the presence of TPs in other muscles (e.g., the sternocleidomastoid) and the emergence of new TPs in the region.

Although the present study investigated the isolated effect of LLLT, the observed improvement in neck disability suggests that this intervention may have potential as a complement to other therapeutic approaches commonly used in clinical practice. Future studies should investigate the long-term effects of LLLT, determine whether its combination with active interventions is beneficial, and perform continuous monitoring of TP evolution throughout the intervention protocol.

As limitations, this study did not assess participants’ treatment expectations or pain catastrophizing levels. Patients with higher catastrophizing tendencies often believe that their pain will inevitably worsen and that their ability to cope is limited, which can negatively affect treatment response [37]. Moreover, previous exposure to LLLT was not assessed and therefore was not considered as an exclusion criterion. Additionally, although participant blinding procedures were carefully implemented, the success of blinding was not formally evaluated.

The method used to assess Tsk may also have introduced measurement bias, suggesting that future research should use a more localized “spot” analysis to detect microcirculatory changes that might be masked by a larger ROI. The adoption of a fixed method for assessing TPs may also be considered a limitation, as, while treatment may change the status or resolve trigger points [13], factors such as lifestyle and psychological factors may promote the development of other trigger points [38].

Additionally, the follow-up period, restricted to the first 48 h after intervention, may have been insufficient to fully capture the therapeutic effects of LLLT, especially considering its potential cumulative effects and the chronic nature of MPS. Future studies should investigate both long-term effects (4 to 12 weeks) to capture the cumulative nature of LLLT and should adopt more pragmatic designs that may investigate whether periodic reassessment and redirection of treatment to newly identified trigger points throughout follow-up influence clinical outcomes.

## Conclusion

The findings of this study suggest that LLLT was safe under the conditions tested; however, its effects at a dosage of 16 J were limited with respect to pain intensity, number of TPs, Tsk, and global perceived effect. These results indicate restricted functional impact within the scope of the present protocol and highlight the need for further investigation using different treatment protocols, a greater number of sessions, and longer follow-up periods. 

## Data Availability

The datasets generated and/or analyzed during the current study are available in the Mendeley repository at 10.17632/y86rwg29j9.1.
